# Addressing cataract in rural Malawi: the Nkhoma Eye Programme

**Published:** 2014

**Authors:** William H Dean, Justin C Sherwin, Ephraim Kambewa, Nick H Metcalfe

**Affiliations:** CBM Ophthalmologist: Nkhoma Eye Hospital, Nkhoma, Malawi. Email: whd1_uk@hotmail.com; Ophthalmology Registrar: Royal Victorian Eye and Ear Hospital, Melbourne, Australia.; Ophthalmic Clinical Officer Cataract Surgeon: Nkhoma Eye Hospital, Nkhoma, Malawi.; CBM Ophthalmologist: Nkhoma Eye Hospital, Nkhoma, Malawi.

**Figure:**
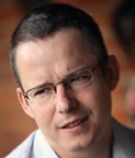
William H Dean

**Figure:**
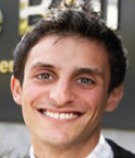
Justin C Sherwin

**Figure:**
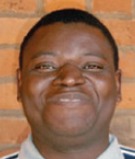
Ephraim Kambewa

**Figure:**
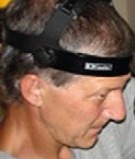
Nick H Metcalfe

Malawi has four main eye care centres for its population of 14.8 million. The eye unit based at Nkhoma Eye Hospital opened in 1955, and CBM has been supporting it since 1977. The hospital is the home of the Nkhoma Eye Programme (NEP), which was started as a VISION 2020 district programme in 2000. In cooperation with the Malawi Ministry of Health (MOH) and other, non-governmental, organisations, the programme provides eye care services in central-western and central-eastern Malawi (population 4.5 million). Cataract operations are performed by an ophthalmologist and a clinical officer who trained as a cataract surgeon.

In 1999, the prevalence of blindness (visual acuity [VA] <3/60 in the best eye) in people aged 40 years or older in Nkhoma was estimated at 3.7%, of which 62% was due to cataract.^1^ Only one in seven people who were blind from cataract and living within 10 miles of Nkhoma had been operated on. A survey conducted seven years later, in 2006, estimated the prevalence of blindness at 1.3%^2^, of which 36% was due to cataract.^3^ By then, four out of every five people who were blind from cataract and living within 10 miles of Nkhoma had been operated on.

Here, we discuss the strategies that led to improved management of cataract.

## Case finding

Since 2003, NEP has been involved in the screening of over 30,000 people per year for cataract. Only 5% of people self-refer. NEP uses three methods for case finding.

- Three community member, employed by NEP, run daily clinics to screen for cataracts in designated districts and villages (25% of case finding).
- Eight mobile eye clinics, run by NEP, visit villages in the catchment area according to a well-publicised schedule (35% of case finding).
- NEP cooperates closely with the Malawi Council of the Handicapped (MACOHA), whose community-based health care workers assist with case finding (30–40% of case finding).

## Quality of surgery

Since 2004, all operations have been performed using a sutureless technique. The quality of surgery is high, and all outcomes are prospectively monitored. Approximately 90% of operations result in a good outcome (VA≥6/18) following correction. Less than 2% have a poor outcome (VA<6/60).

## Increased surgical output

The number of cataract operations performed per year has risen from just over 400 in 1999 to over 4,000 in both 2008 and 2009 – a ten-fold increase in ten years (Figure 1).

**Figure:**
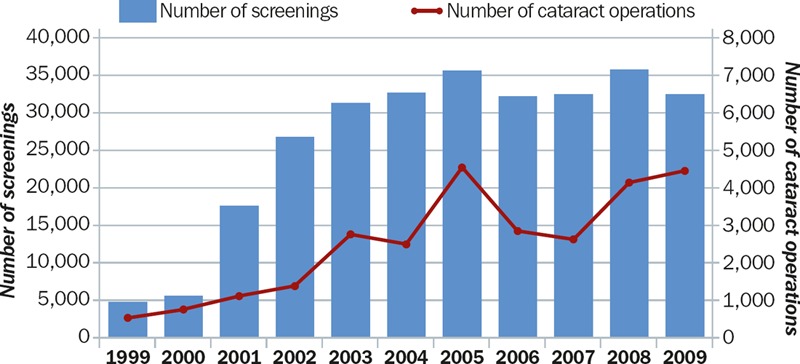
Figure 1. Management of cataract with Nkhoma Eye Programme (1999–2009)

**Figure:**
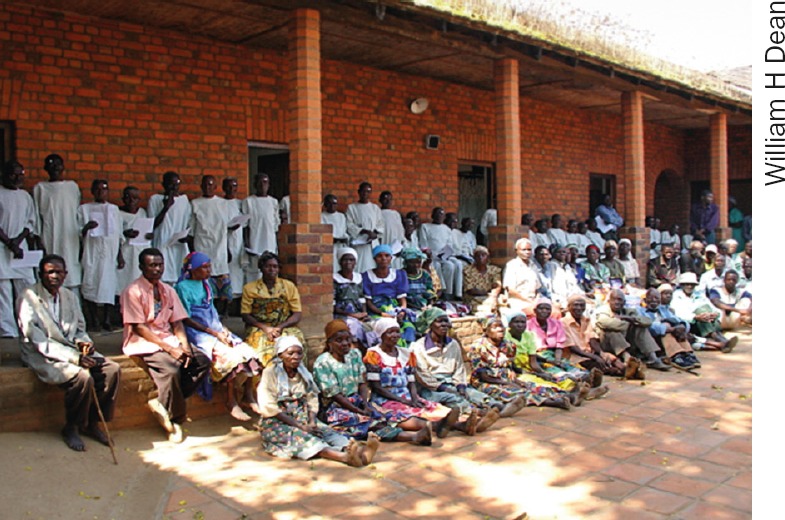
Patients waiting for surgery at Nkhoma

There also have been significant increases in cataract surgical output since the initiation of VISION 2020 programmes in two districts in East Africa (Kwale in Kenya, and Kilimanjoro in Tanzania).^4^ Similarities between Nkhoma and these districts include:

- assistance with transport
- free examinations
- close links between the hospital and community services
- minimal waiting times before surgery.

Additional factors at NEP include:

- active case finding
- training of ophthalmic clinical officers to perform cataract surgery
- surgical outreach
- infrastructure development
- Support from national and international partners
- Patient satisfaction with post-operative outcomes and rehabilitation, which has led to a good reputation in the community
- Strong links with traditional authorities, village headmen and chiefs who assist in mobilising their communities, selecting volunteers and promoting our services.

One Nkhoma ophthalmologist and the MACOHA coordinator have permanent positions on the National Committee for the Prevention of Blindness (NCPB). Cooperation with the MOH and other stakeholders ensures that limited resources are maximised. The NEP and MACOHA are fully integrated into the Malawi National VISION 2020 plan.

NEP has improved its eye care services over the last decade. An emphasis on continual improvement and the development of trained ophthalmic staff will help to ensure sustainability in eye care delivery in the future.
